# A GeoAI framework for detecting risk zones from illegal dumping sites in Khulna, Bangladesh

**DOI:** 10.1371/journal.pone.0345178

**Published:** 2026-03-18

**Authors:** Swadhin Das, Md. Manjur Morshed

**Affiliations:** Department of Urban and Regional Planning, Khulna University of Engineering & Technology (KUET), Khulna, Bangladesh; University of Limpopo, SOUTH AFRICA

## Abstract

Illegal dumping is a major challenge of municipal solid waste management. A significant portion of waste is dumped illegally in cities worldwide. This improper waste disposal creates serious ecological degradation, environmental hazards, public health risks, and urban planning challenges. This study presents a Positive-Unlabeled (PU) learning framework for detecting illegal dumping sites using GeoAI. Seven machine learning algorithms were trained on 70% of 341 confirmed illegal dumping sites across 10 m × 10 m resolution grids covering Khulna City Corporation, Bangladesh. The remaining 30% of the data was reserved for independent testing. The best model was selected using a weighted composite score combining the Area Under the Curve (AUC) and F1 score from spatial cross-validation, and the selected best model was subsequently evaluated on an independent hold-out test set. Random Forest achieved the highest AUC (0.883) and F1 score (0.820), and consistently outperformed other models across all weighting schemes. The three most influential predictors of illegal dumping were proximity to roads, drains, and buildings. The predicted risk map shows that the very high and high-risk zones are concentrated along roads and urban centers. This study introduces the first GeoAI framework for illegal dumping site detection in Khulna, Bangladesh. This framework can also be applied in other cities to detect illegal dumping sites at the community level. This methodology can help municipal authorities to develop a waste management plan that addresses both illegal dumping challenges and long-term infrastructure planning.

## Introduction

Illegal dumping of municipal solid waste (MSW) remains a key challenge for city authorities. The term “illegal dumping” refers to the disposal of waste in unauthorized public or private places [1,2]. Globally, the amount of waste illegally dumped annually is estimated to reach close to 100 million tons [3]. Some of the primary factors in the rise of illegal dumping sites (IDS) include growing populations, rapid urbanization, lack of awareness, consumerism, and a lack of waste management infrastructure [4,5]. Waste is illegally dumped for numerous reasons, such as avoiding fees, saving on travel time and expense, unavailability of collection service, unwillingness to dispose of the waste properly, or ignorance [1,2,6]. In many developing countries, up to 60% of waste remains uncollected [7], and a large portion of the uncollected waste ends up in illegal dumps, water bodies, or open spaces [8].

Illegal dumping is not just a nuisance, but it may also lead to numerous additional difficulties [1,9]. It has become prevalent in many urban areas and poses significant environmental and public health risks [10]. The quality of life and livability of the surrounding communities are significantly impacted by IDSs [11]. The ecosystem has been negatively impacted by illegal dumping, which exposes people’s health and well-being to severe risk [5,12,13]. Illegal dumping can contaminate groundwater and other water sources, obstructing flow and resulting in floods [11]. Additionally, it creates breeding grounds for disease-carrying rats and mosquitoes [14]. Therefore, detecting and monitoring illegal dumping is crucial for environmental protection and public health.

Bangladesh faces severe challenges with illegal dumping due to its high population density and rapid urbanization. Illegal dumps often contain plastic debris, which not only degrades landscapes but also poses ecological toxicity. A recent global meta-analysis demonstrated that micro- and nano plastics significantly impair insect survival and growth, indicating long-term biodiversity risks associated with unmanaged waste [15]. The situation exemplifies the waste management challenges faced by cities in developing countries. The environmental conditions, socioeconomic factors, and existing waste management infrastructure in Bangladesh create a unique context that necessitates locally adapted solutions.

Historically, the public reporting of illegal disposal activities has contributed to the detection of IDS [1]. Still, there are concerns about how effective this method is. Traditional methods are often labor-intensive and inefficient [16]. While remote sensing techniques have developed as a powerful tool for environmental monitoring [17], their application to IDS detection remains limited. Remote sensors can be used to discriminate amongst illegal dumping based on their distinctive spectral reflectance [18]. Satellite imagery analysis combined with machine learning (ML) algorithms offers promising approaches for identifying potential IDSs.

Globally, ML has proven effective in optimizing environmental monitoring and inspection strategies by enabling data-driven identification of high-risk sites [19]. ML has also been successfully applied to quantitative risk assessment in safety-critical systems, demonstrating its potential to support spatial risk prediction in urban environments [20]. Various ML models such as Random Forest (RF), K-Nearest Neighbors (KNN), Multilayer Perceptron (MLP), Logistic Regression (LR), Support Vector Machine (SVM), Decision Tree (DT), and Naive Bayes (NB) have demonstrated effectiveness in environmental classification and can be used for IDS detection [21–25].

Scientific approaches to detect IDS have advanced from reactive enforcement to proactive prevention strategies. Matsumoto and Takeuchi (2011) proposed a framework for addressing illegal dumping that combines improved waste collection services, affordable disposal options, law enforcement, and public education campaigns [10]. GIS and remote sensing techniques have proven as valuable tools for identifying and monitoring IDS. Similar approaches have been employed in many countries to create illegal dumping risk maps [22]. Community-based monitoring programs have also shown potential in addressing illegal dumping. A successful citizen science program was reported in Australia, where volunteers used mobile applications to report IDSs [16].

Previous studies explored various factors influencing illegal dumping using different methods. These factors include socio-economic [26–29], environmental [5,30], waste management infrastructure [5,30,31], social norms [26,29,31], government policies [29,31,32], geographic characteristics [26,27,30,33] etc. The researchers used diverse analytical methods, such as multinomial logit models [26], GIS analysis [30,33], spatial regression [27,28], ISM-DEMATEL [31], etc. Key analytical approaches include remote sensing techniques, spatial analysis, statistical modeling, and community-based research methods [28,29].

Despite these advances, a significant gap remains in the literature regarding the application of integrated ML, GIS, and remote sensing (RS) approaches for IDS detection in the context of rapidly growing cities in Bangladesh. For example, Karimi et al. (2022) developed a framework for mapping IDS, which is more specifically applicable for rural and remote areas [5]. Geng et al. (2024) divided the study area into 1 km × 1 km grid, which is suitable for large scale assessments [22]. However, cities like Khulna need a more detailed evaluation because waste management facilities and illegal dumping activities occur at the community level. For example, the Rajbandh landfill in Khulna is less than 1 km². Therefore, this study implements a 10 m × 10 m grid to analyze IDS patterns more effectively. Compared to previous studies [5,22,30,33–35], this study uses a broader and more diverse set of predictors.

The objective of this study is to develop and validate a GeoAI framework for detecting IDS in Khulna, Bangladesh. This study addresses several specific research questions. It examines the spatial patterns of IDS in Khulna City, explores which ML algorithms provide the most accurate detection of IDS in the Bangladeshi context, and identifies what factors correlate most with IDSs in Khulna. Methodologically, this study uses a multi-stage approach that combines field surveys of existing IDSs, remote sensing data analysis, geospatial modeling and ML algorithms. There has been no study that uses GeoAI for detecting IDSs in Khulna city. The outcomes of this study have the potential to contribute significantly to improving waste management practices in Khulna City Corporation (KCC). By developing a framework that integrates ML, GIS and RS, the study aims to provide actionable insights that can guide decision-makers in detecting IDSs.

## Materials and methods

### Study area and data

The study area for this research is KCC. Khulna city is the third largest city in Bangladesh and its municipality’s (KCC) projected population is 1.3 million [36,37]. The area of KCC is 45.65 square kilometers, situated between latitudes 24°45′-24°54′ north and longitudes 89°28′-89°35′ east. Khulna’s climate is classified as tropical wet and dry or savanna under the Köppen classification system. Temperatures remain warm throughout the year (26.5°C to 36.2°C) with heavy rainfall during monsoon periods [38]. The monsoon period lasts from mid-June to mid-August [39].

MSW generation in the city has increased rapidly, from 520 tons per day in 2005 to 1000 tons per day in 2023 [40,41]. The household waste generation rate of Khulna is 0.472 kg/c/d. About 81% of waste generated in households is biodegradable [42]. Waste from various sources is collected by NGOs and CBOs and transported to nearby Secondary Transfer Station (STS) [43], where KCC trucks then carry it to Rajbandh landfill [44]. KCC estimates around 80% of the daily generated MSW is collected [36,41]. The remaining 20% of MSW is never dumped in landfills for various reasons and they end up being dumped at different unauthorized places, creating IDSs. This waste is often dropped at the roadside or open drains by city dwellers. The city faces waterlogging during the rainy season [45]. Inadequate drainage systems and poor waste disposal cause drain clogging and waterlogging [46].

Initially, 15 predictor variables were selected based on their potential influence on IDS patterns. The socio-economic aspects include population density, livelihood index, poverty index, infrastructure index and informal settlements. Geographic and spatial factors incorporate digital elevation model (DEM), drains, buildings, rail lines, roads, road intersections and water bodies. The environmental component focuses on land surface temperature (LST), while waste management factors are represented by the household waste generation rate and the locations of STSs where waste is temporarily stored. These predictors were carefully selected based on a thorough literature review to understand their relationship with illegal dumping behavior in the context of Khulna. Previous studies have demonstrated that these factors are associated with illegal dumping activities [5,22,26–30,33,47–59]. However, criteria related to awareness and law enforcement have been excluded because of their uniform nature across the city. Multicollinearity was assessed using Variance Inflation Factors (VIF). Three predictors (informal settlements, livelihood index, and infrastructure index) were removed because of VIF values exceeding the threshold of 10. Table 1 contains the final VIF values after removal.

**Table 1:** Description of spatial datasets and multicollinearity.

Data	Year	Type	VIF	Source
Illegal Dumping Site (IDS)	2024	Vector data	–	Survey
Population density	2024	Raster Data	1.55	KCC
Poverty index	2020	Raster Data	2.49	SNV
Secondary Transfer Station (STS)	2024	Vector data	1.56	SCIP
Road Intersection	2024	Vector data	2.25	Open Street Map
Rail Line	2024	Vector data	1.33	Open Street Map
Road	2024	Vector data	2.27	Open Street Map
Drainage	2024	Vector data	1.98	KCC
Waterbody	2024	Vector data	1.07	Open Street Map
Buildings	2024	Vector data	1.55	Open Street Map
Land Surface Temperature (LST)	2024	Raster Data	2.74	GEE
DEM	2024	Raster data	1.39	Copernicus DEM
Household Waste Generation Rate	2023	Vector data	2.63	SCIP

The locations of 341 IDSs were identified through a combination of field surveys, satellite images, Google Earth Street View, and GPS surveys. No specific permits were required for this study because the fieldwork was conducted in publicly accessible areas. Population and drainage data were obtained from KCC for 2024. The poverty index was collected from a report of SNV. Several geographic features like buildings, railway lines, roads, road intersections, and water bodies were extracted from OpenStreetMap (2024). LST data was accessed through the Google Earth Engine (GEE). Data about STSs and household waste generation rate was provided by the SCIP Plastics Project. A digital elevation model (DEM) was obtained from the Copernicus DEM dataset with a spatial resolution of 30m. All datasets were collected and analyzed according to data-use policies of each data provider.

### Data processing and model implementation

All datasets were processed to ensure compatibility (reprojection to a common coordinate reference system (WGS84), repair of invalid geometries and establishment of a consistent spatial extent). Distance from each cell to vector features was derived using the Distance Accumulation tool of ArcGIS Pro. The study area was divided into a 10 m × 10 m grid. The full dataset contains 454,558 grid cells. All predictor variables were extracted by mean values at the centroid of each grid cell. Table 2 represents the sample structure of the dataset used for model development. Missing values in the predictor datasets were imputed using nearest-neighbor interpolation. Any remaining missing values were subsequently filled using global column means.

**Table 2:** Sample structure of the PU training dataset used for model development.

Grid ID	Status	Population density (persons/km²)	DEM (m)	Household Waste Generation Rate (kg/capita/day)	Poverty Index	LST (°C)	Distance to the nearest drain (m)	Distance to the nearest building (m)	Distance to the nearest rail line (m)	Distance to the nearest road (m)	Distance to the nearest road intersection (m)	Distance to the nearest waterbody (m)	Distance to the nearest STS (m)
439351	Positive	17950	5.7	0.37	65	33.79	1	13.89	32.65	1	159.24	123.14	270.8
4295	Unlabeled	14798.4	9.53	0.38	62	33.16	1282.1	35.17	5346.81	53.94	74.17	14.68	854.38
446074	Positive	14833.4	9.8	0.43	53	33.95	4.12	31.05	545.74	208.27	255.19	140.3	364.03
59259	Unlabeled	64423.4	8.18	0.46	60	35.9	4	2.24	2305.68	3	49.45	82.19	616.36
379019	Unlabeled	10294.5	8.2	0.4	71	34.54	17.26	6.32	641.31	26.31	34.11	39.7	648.5

**Fig 1:**
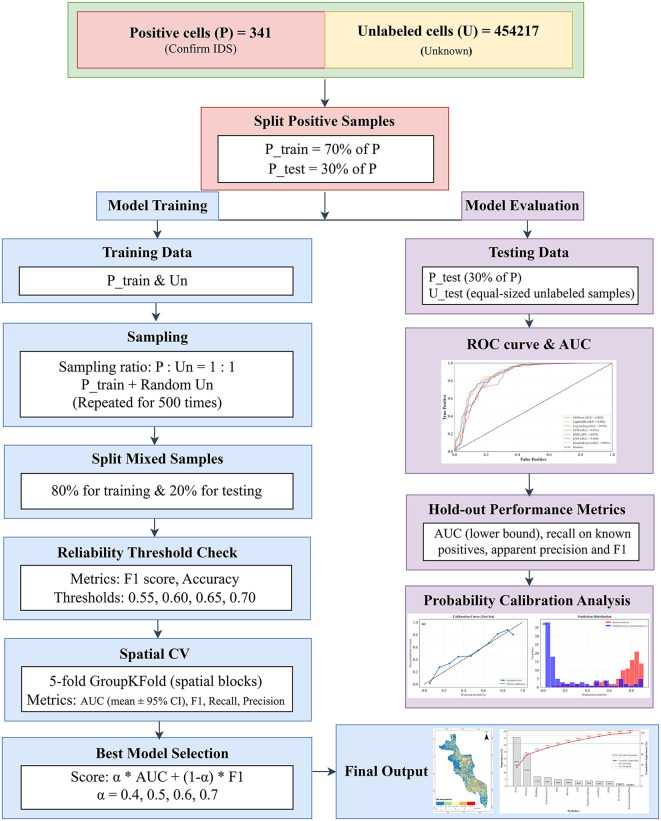
Workflow of the PU learning framework for illegal dump risk mapping.

The grid cells were categorized into two sets. The Positive set (P) includes 341 confirmed IDS locations, whereas the Unlabeled set (U) contains 454,217 cells where the dumping status is unknown (Fig 1). Positive samples were divided into P_train (70%) and P_test (30%) subsets before any model training or hyperparameter tuning to prevent data leakage. An equal number of unlabeled samples were randomly selected for testing to ensure balanced evaluation. Predictor variables were processed differently for tree-based models and linear/kernel-based models. Raw distance features were retained for tree-based models due to their ability to handle nonlinear relationships. On the other hand, logarithmic transformations to distance variables were applied for linear/kernel-based models to stabilize variance. All features were standardized to ensure comparable scales across algorithms. Spatial blocks were created using k-means clustering on centroid coordinates (k = 5). In each fold, models were trained on four spatial blocks and validated on the remaining block.

To address the class imbalance and the uncertainty in the unlabeled data, a PU bagging approach with 500 bootstrap iterations was used with spatial block cross-validation. Preliminary convergence analysis showed that model performance metrics stabilized well before 500 iterations. No noticeable improvement was observed beyond this point. Therefore, 500 iterations were selected to ensure robust probability estimation. In each iteration, a balanced training dataset was created by pairing all positive samples (P_train) with an equal number of randomly sampled unlabeled data (Un). This technique mitigates the bias that would result from treating all unlabeled cases as negative classes. Within each iteration, the balanced training dataset was further split into 80% for training and 20% for validation. This internal validation set was used to assess classifier reliability through F1-score and accuracy. A sensitivity analysis was conducted across four reliability thresholds (0.55, 0.60, 0.65, and 0.70) to assess the trade-off between ensemble size and classifier quality. Only classifiers that met minimum reliability thresholds (0.55–0.70) were retained for the final ensemble.

Seven ML models (KNN, RF, MLP, LR, SVM, XGBoost, and LightGBM) were used for IDS detection. This selection includes diverse computational paradigms within ML (Table 3). KNN is ideal for spatial data because it recognizes patterns based on proximity, which is essential when locating IDSs. For a location p, the distance to other known locations considering all factors (Equation 1):

**Table 3:** ML models and corresponding hyperparameter ranges used for tuning.

Model	Category	Hyperparameters	Tested values	Best values
Random Forest (RF)	Ensemble	n_estimators	200, 500	200
		max_depth	10, 20, None	20
		min_samples_split	5, 10	10
		min_samples_leaf	2, 4	4
		max_features	sqrt, log2	sqrt
K-Nearest Neighbors (KNN)	Instance-based	n_neighbors	5, 9, 15, 21	21
		Weights	uniform, distance	uniform
		metric	euclidean, manhattan	manhattan
		p	1, 2	1
Multilayer Perceptron (MLP)	Neural Network	hidden_layer_sizes	(100), (100, 50), (150, 75, 25)	(150, 75, 25)
		activation	relu, tanh	tanh
		alpha	0.0001, 0.001, 0.01	0.0001
		learning_rate	adaptive	adaptive
Logistic Regression (LR)	Linear Probabilistic	C	0.01, 0.1, 1, 10	0.1
		penalty	l2	l2
		solver	lbfgs	lbfgs
Support Vector Machine (SVM)	Margin-based	C	0.1, 1, 10, 100	1
		kernel	Rbf, poly	rbf
		gamma	scale, 0.01, 0.1	scale
eXtreme Gradient Boosting (XGBoost)	Ensemble	n_estimators	100, 300	100
		max_depth	3, 5, 7	3
		learning rate	0.01, 0.05, 0.1	0.01
		subsample	0.8, 1	0.8
		colsample_bytree	0.8, 1	0.8
		min_child_weight	1, 3	1
Light Gradient Boosting Machine (LightGBM)	Ensemble	n_estimators	100, 300	100
		max_depth	3, 5, 7	3
		learning_rate	0.01, 0.05, 0.1	0.1
		num_leaves	15, 31, 63	15
		min_child_samples	10, 20	20
		subsample	0.8, 1.0	0.8


$$\begin{array}{c} d\left(p,p_{i}\right)=\sqrt{\sum {\mathrm{\backslash nolimits}}_{f\in factors}{w_{f}(x_{f,p}-x_{f,i})}^{2}} \end{array}$$ (1)

Where w_f_ is the weight for each factor, x_f,p_ is the value of factor f at location p, and x_f,i_ is the value of factor f at the i^th^ location. Prediction of IDS probability (Equation 2):


$$\begin{array}{c} P\;(IDS=1|p)=\;\frac{1}{k}\;\sum {\mathrm{\backslash nolimits}}_{i=1}^{k}I(y_{i}=1) \end{array}$$ (2)

Where p is the location being evaluated, k is the number of nearest neighbours considered, and I(y_i_ = 1) is an indicator function that equals 1 if the i^th^ nearest neighbour is an IDS, 0 otherwise. RF belongs to ensemble methods that aggregate predictions from multiple decision trees. RF works well with large datasets and non-linear connections [60], providing valuable understanding of which factors are most influential (Equation 3).


$$\begin{array}{c} P(IDS=1|X)=\;\frac{1}{M}\;\sum {\mathrm{\backslash nolimits}}_{m=1}^{M}h_{m}(X) \end{array}$$ (3)

Where P (IDS = 1|X) is the probability of an IDS given input features X, M is the total number of trees, h_m_(X) is the prediction of the m^th^ tree, and X is the vector of input features. MLP functions as a neural network architecture with interconnected layers of nodes. MLP is admirable at understanding complicated patterns and interactions, making it perfect for non-linear relationships (Equation 4).


$$\begin{array}{c} h_{1}=\sigma (\sum {\mathrm{\backslash nolimits}}_{f\in factors}w_{1f}x_{f}+b_{1}) \end{array}$$ (4)

$$P\;(IDS=1|X)=\sigma (w_{2}h_{1}+b_{2})$$ (5)

Where h_1_ is the hidden layer output, w_1f_ is weights for each factor, x_f_ is input features, b_1_, and b_2_ are bias terms, and σ is the sigmoid activation function (Equation 5). LR provides a probabilistic framework for binary classification of locations as potential illegal dump sites through linear combination of input features (Equation 6).


$$P\;\left(IDS=1|X\right)=\;\frac{1}{1+\text{exp}\left[-\left(\beta_{o}+\sum_{i=1}^{12}\beta_{i}X_{i}\right)\right]}$$ (6)

Where, β_o_ is the intercept, β_i_ is the coefficient for predictor X_i_ and X_i_ is the 12 spatial, socio-economic, environmental, and infrastructure-related factors. SVM performs margin-based classification by identifying optimal hyperplanes that separate dump sites from non-dump sites in high-dimensional space (Equation 7).


$$P\;\left(IDS=1\;|\;x\right)=\;\frac{1}{1+\text{exp}(A\left[\sum_{j=1}^{N}a_{j}y_{j}K\left(x_{j},x\right)+b\right]+B)}\;,\;x\in R^{12}$$ (7)

Where, x denotes the 12 predictor vector, a_j_ and y_j_ are support-vector weights and labels, K(.) is the kernel function, b is the bias term, and A, B are Platt scaling parameters. XGBoost and LightGBM are ensemble learning methods based on gradient boosting that generates trees in successive iterations (Equation 8).


$$\begin{array}{c} P(IDS=1|X=\sigma (\sum {\mathrm{\backslash nolimits}}_{t=1}^{T}f_{t}(X)) \end{array}$$ (8)

Where, T is the total number of trees, f_t_(X) is the t_th_ boosted tree prediction, and σ is the sigmoid activation function. Hyperparameter tuning was conducted using GridSearchCV with spatial cross-validation (5-fold GroupKFold) on a balanced training subset (Table 3).

Model evaluation was conducted in two stages. First, spatial cross-validation (5-fold GroupKFold) was performed on the training dataset to compare model performance and select the best model. Multiple metrics including accuracy, precision, recall, F1-score, and AUC were computed across folds. Accuracy reflects the ratio of samples classified correctly out of all samples (Equation 9).


$$\begin{array}{c} Accuracy(f;D)=\frac{1}{n}\sum {\mathrm{\backslash nolimits}}_{i=1}^{n}I(f(x_{i})=y_{i}) \end{array}$$ (9)

Where D is the data set; n is the number of samples; I is an indicator function and the value of I is 1 when the corresponding equation is satisfied, otherwise the value of I is 0; f(xi) is the predicted class label; yi is the actual class label. The ratio of correctly identified IDS to all predicted dumping was measured by precision, while the ratio of correctly identified IDSs to all actual dumps was measured by recall. The F1 score offered a well-balanced assessment of model performance by calculating the harmonic mean between precision and recall values. Precision and recall can be expressed as Equation 10 and Equation 11:


$$Precision=\frac{TP}{TP+FP}$$ (10)

$$Recall=\frac{TP}{TP+FN}$$ (11)

TP represents the total number of samples that belong to the actual positive class and are classed as such, whereas FP is the number of samples that belong to the actual negative class but are classified as positive, and FN is the number of samples categorized as negative class but belong to the positive class. F1 score is the harmonic mean of precision and recall as Equations 12:


$$F1=\frac{2\times P\times R}{P+R}$$ (12)

Where *P* is precision, *R* is recall. Model performance was compared using a weighted composite ranking score. Because the dataset is highly imbalanced and the models were trained within a PU learning framework. The F1 score was considered as it reflects the balance between precision and recall. The AUC was included to capture the overall discriminatory capability of each model. The final ranking score was calculated by Equation 13. Where, α ∈ {0.4, 0.5, 0.6, 0.7}.


Score= α ×AUC+(1−α)×F1 (13)

The best-performing model based on these criteria was selected for the final IDS risk map. Then, the hold-out test set was used for final model evaluation. ROC curve, probability calibration, and hold-out performance metrics (AUC, recall on known positives, and apparent precision and F1-score) were calculated using the hold-out test set. Final risk probabilities were computed using weighted averages across all valid classifiers. Feature importance analysis was conducted to identify the most influential factors in IDS prediction.

### Software and tools used

This study used several software and tools to support data collection, processing, and model development. Coordinates – GPS Formatter was used to record the coordinates of illegal dumping sites during field visits and Google Earth Pro (version 7.3.6.9750) was used for verifying dumping locations. ArcGIS Pro (version 3.4) was used to prepare the spatial layers, mapping, and generate the 10 m × 10 m grid. Land Surface Temperature data were processed through Google Earth Engine. Model training, statistical analysis, feature importance analysis, and comparative evaluation of the seven models were performed in a Jupyter Notebook using Python (version 3.13). Key Python libraries included NumPy, pandas, scikit-learn, Matplotlib, and SciPy.

## Results

### Illegal dumping in KCC

**Fig 2:**
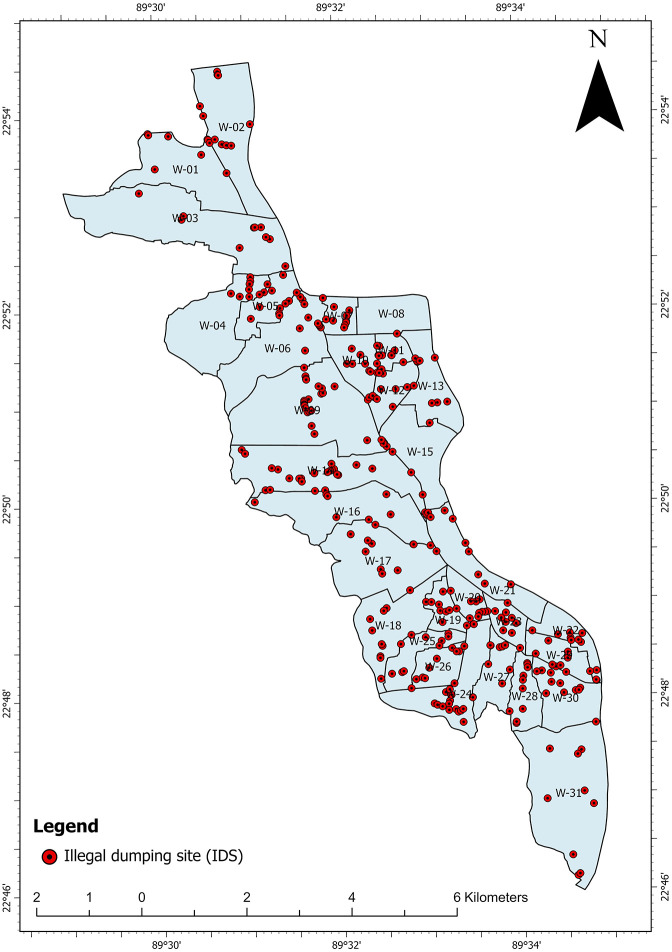
Spatial distribution of IDSs.

A total of 341 IDSs were identified across KCC and used for model training. Fig 2 illustrates the spatial distribution of IDSs across different wards in KCC. These locations were identified through field surveys, Google Earth Street View, GPS surveys, and satellite image analysis. Except for Ward 08 and Ward 15, every ward has at least 10 IDSs. Most of the areas of Ward 08 are currently empty due to the closing of jute industries. There is a Bangladesh Navy base in Ward 15, which is restricted from public access.

### Spatial distribution of predictor variables

**Fig 3:**
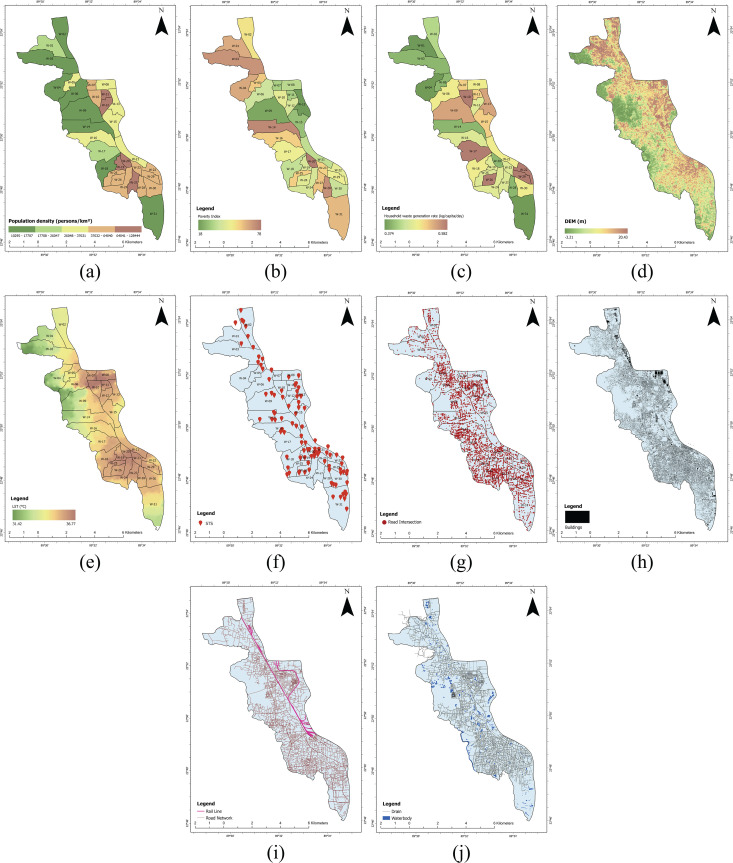
Spatial distribution of (a) population density, (b) poverty index, (c) household waste generation rate, (d) DEM, (e) LST, (f) STS, (g) road intersection, (h) buildings, (i) rail and road, (j) drain and waterbody.

Fig 3(a) shows population density across different wards of KCC. Ward 12 has the highest population of 88,745 residents despite its relatively small area of 0.69 square kilometers. Ward 04 has the lowest population with 24,381 residents. On the other hand, some larger wards like Ward 03 and Ward 31 show more moderate population density. The poverty index values across the wards range from 18 to 78 (Fig 3(b)). Ward 20 demonstrates the highest poverty index at 78, followed by Ward 14 (74) and Ward 28 (72). On the other hand, Ward 13 shows the lowest poverty index at 18. Fig 3(c) illustrates ward-wise variation in household waste generation rates in KCC. Fig 3(d) shows the spatial variation of elevation across KCC.

Fig 3(e) depicts the spatial distribution of LST across KCC in 2024. The temperature ranges from 31.42°C to 36.23°C. Higher temperatures are concentrated in the eastern and southeastern parts of KCC. In KCC, there are 100 STSs designated for temporarily storing MSW (Fig 3(f)). These STSs serve as intermediate storage points for waste before it is transported to the landfill site for final disposal. Fig 3(g) shows the spatial distribution of road intersections across KCC. There are 4332 road nodes throughout the city. Fig 3(h) illustrates the spatial distribution of settlements in KCC. Fig 3(i) displays the spatial distribution of road and rail lines in KCC. The total length of the road network in KCC is approximately 570 kilometers. Fig 3(j) shows the spatial distribution of the drain and waterbody in KCC covering a total area of 1.55 square kilometers.

### Descriptive statistics of predictor variables

Table 4 depicts the descriptive statistics of the predictor variables. The study area shows significant variability across all variables. Population density is very high, with a mean of 29,055 persons per km². The moderate dispersion of poverty, infrastructure, and livelihood indices indicates the heterogeneous socioeconomic conditions across the city. Distances to informal settlements, drainage networks, water bodies, and secondary transfer stations (STS) show comparatively higher standard deviations. LST and DEM exhibit comparatively low variability. The rate of household waste generation is quite stable across the study area (SD = 0.07).

**Table 4:** Descriptive statistics of the predictor variables.

Variables	Mean	Median	Min	Max	SD
Population density (persons/km²)	29055.1	17950	10294.5	128444	21781.28
Poverty Index	50.02	53	18	78	16.85
Distance to the nearest STS (m)	492.19	363	1	2747.89	427.56
Distance to the nearest road intersection (m)	85.03	59.40	1	811.60	87.53
Distance to the nearest rail line (m)	1426.64	1156.13	1	6224.35	1252.05
Distance to the nearest road (m)	50.06	26.17	1	776.96	73.37
Distance to the nearest drain (m)	112.51	28.64	1	1911.96	228.28
Distance to the nearest waterbody (m)	208.37	137.32	0.70	1906.35	243.65
Distance to the nearest building (m)	25.58	12.04	1	406.74	39.55
LST (°C)	34.70	34.92	31.43	36.77	1.15
DEM (m)	6.04	6.08	−3.22	20.43	2.74
Household Waste Generation Rate (kg/capita/day)	0.46	0.46	0.37	0.58	0.07

### Model evaluation and selection

#### Spatial cross-validation performance.

Table 5 presents the comparative performance of seven models using different evaluation metrics. The listed values in Table 5 represent the mean results obtained from spatial cross-validation folds. Overall, all models achieve high AUC values (0.862–0.883). The RF model demonstrates a well balanced performance across all evaluation metrics. RF achieves the highest AUC (0.883) and F1 (0.820), as well as a balanced accuracy, precision, and recall. On the other hand, KNN has the lowest accuracy (0.767) and F1 (0.768). Though, MLP achieves the highest recall (0.871), it has lower precision and F1 score. LR demonstrates high precision (0.80) but comparatively lower recall (0.801). SVM and XGBoost perform consistently across all metrics. Though their F1 scores remain lower than RF. LightGBM achieves the highest accuracy (0.830) but has lower recall and the lowest F1 score (0.762).

**Table 5:** Performance comparison of ML models.

Model	Accuracy	Precision	Recall	F1 score	AUC
RF	0.816	0.794	0.853	0.820	0.883
KNN	0.767	0.741	0.821	0.768	0.862
MLP	0.796	0.741	0.871	0.794	0.874
LR	0.806	0.80	0.801	0.797	0.875
SVM	0.796	0.775	0.812	0.788	0.878
XGBoost	0.806	0.782	0.846	0.811	0.876
LightGBM	0.830	0.767	0.768	0.762	0.867

#### Model selection through composite ranking.

Table 6 presents the best model across different weighting schemes. The result shows that RF consistently ranks as the best model across all weighting schemes. The performance of RF is not sensitive to the choice of evaluation weights. That’s why RF was selected as the final model and its weighted ensemble predictions were used for risk mapping.

**Table 6:** Model ranking across different weighting schemes.

F1	AUC	Best model
0.4	0.6	RF
0.5	0.5	RF
0.6	0.4	RF
0.7	0.3	RF

#### Hold-out test set evaluation.

**Fig 4:**
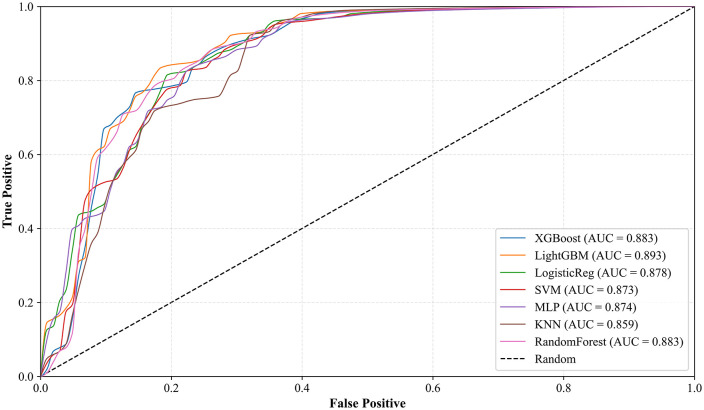
ROC curves for seven models.

Fig 4 illustrates ROC curves for seven ML models evaluated on the holdout test set. The AUC values range from 0.859 to 0.893. LightGBM has the highest AUC (0.893). RF and XGBoost achieved the second highest AUC (0.883). LR, SVM, and MLP show slightly lower but still acceptable performance. While, KNN has the lowest AUC (0.859).

Table 7 was generated to further evaluate the selected model’s performance on the independent test set. The model achieved an AUC of 0.883 and recall of 0.844. The apparent precision (0.798) and F1-score (0.820) represent conservative estimates due to potential positive IDS in the unlabeled test samples.

**Table 7:** Hold-out test set performance of RF.

Metric	Value
AUC (lower bound)	0.883
Recall on known positives	0.844
Apparent precision	0.798
Apparent F1	0.820

#### Probability calibration analysis.

**Fig 5:**
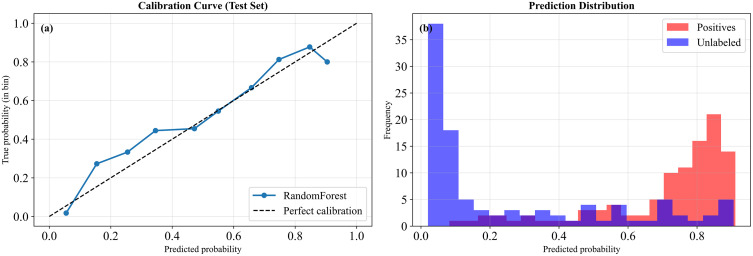
(a) Probability calibration curve and (b) prediction distribution of RF.

Fig 5(a) shows the calibration curve comparing predicted probabilities with observed frequencies. Probability estimates of RF closely follow the perfect calibration line. So, the predicted risk values are well aligned with observed outcomes and suitable for probability-based interpretation. Fig 5(b) displays the distribution of predicted probabilities for known IDSs and unlabeled locations. The prediction distribution shows a clear separation between known IDSs and unlabeled locations. Most of the IDSs have high predicted probabilities, while most unlabeled cells have low predicted probabilities.

#### IDS risk mapping.

**Fig 6:**
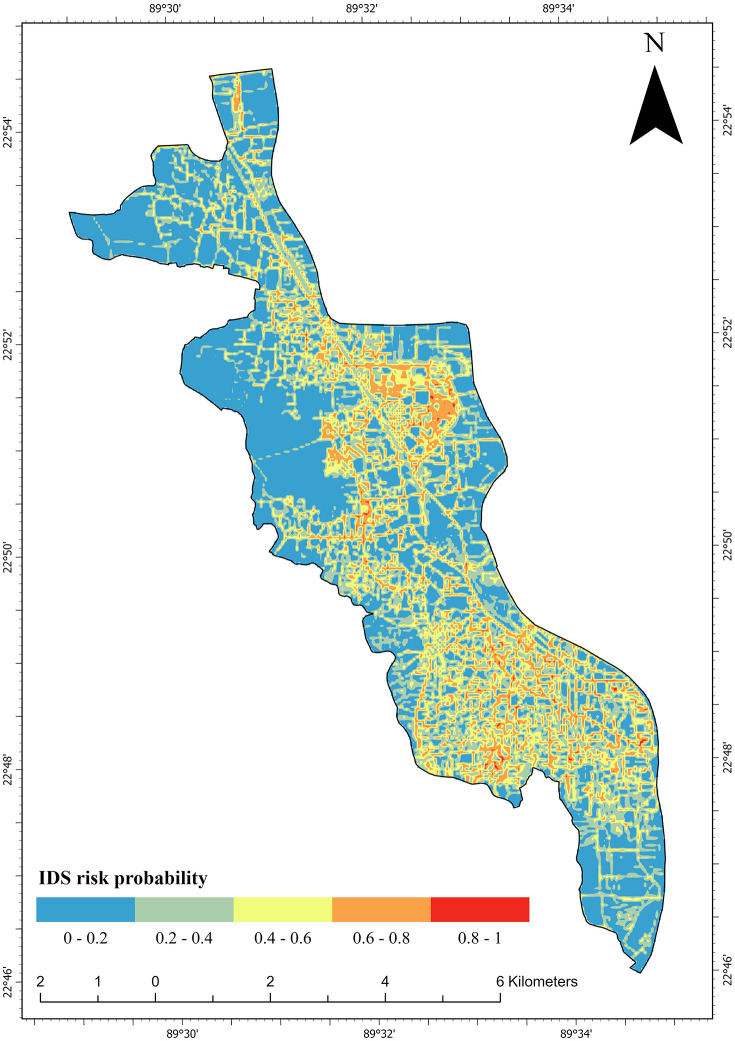
Illegal dump risk score across KCC.

Fig 6 illustrates the spatial distribution of illegal dump risk levels across KCC predicted by the RF model. The spatial distribution of risk exhibits the very high and high-risk zones are concentrated along road networks and urban centers. While the lowest-risk classifications (0–0.2) are found in the peripheral areas. This distribution demonstrates that IDS practice is strongly clustered in highly accessible and densely built areas of the city.

#### Predictor importance analysis.

**Fig 7:**
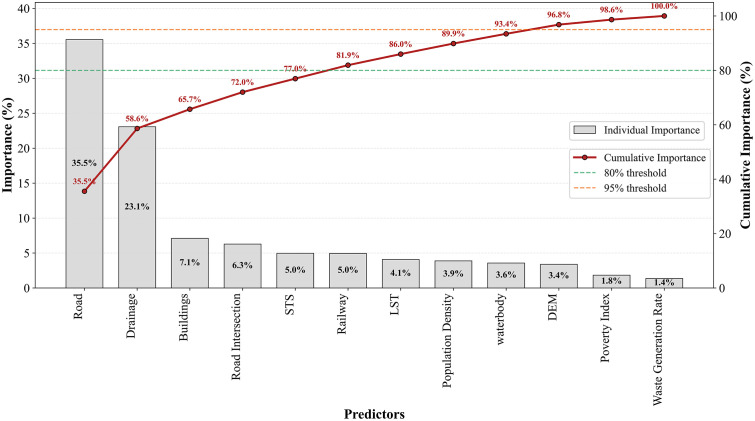
Predictor importance analysis.

Fig 7 presents the importance of predictor variables used in identifying IDS using RF. Road is the most important predictor (35.5%). Drain ranks as the second most important predictor (23.1%).

Road intersections (6.3%) also demonstrate how the transportation network influences dumping behaviors. The importance of rail proximity (5.0%) indicates that linear transportation infrastructure can also influence IDS. Population density (3.9%) shows limited influence. On the other hand, the poverty index (1.8%) and waste generation rate (1.4%) are the least important predictors. Although population density and poverty index vary significantly at ward level, they do not align strongly with IDS at the local scale. Waste is usually dumped illegally where access is easy and waste can be dumped with minimal effort. The top six features account for 81.9% of cumulative feature importance, while the top 10 predictors collectively explained 96.8% of the variance in IDS patterns.

## Discussion

MSW management presents substantial challenges globally, particularly in developing countries. Bangladesh also faces severe challenges in managing MSW effectively. In Khulna, approximately 80% of daily generated MSW is collected, while the remaining 20% is dumped illegally [41]. Improper waste disposal, especially IDS, poses a significant environmental and public health threat [12,61,62]. The findings of this study provide comprehensive insights into the influencing factors of IDS. The spatial distribution of 341 IDSs suggests that it is not only an isolated behavior but also influenced by various factors. The observed patterns in Khulna align with previous research by Matsumoto and Takeuchi (2011), who found that IDS tends to be concentrated in certain areas based on accessibility and surveillance factors [10]. Similarly, Webb et al. (2006) have identified that accessibility is often a key determinant of IDS locations [63].

The predictor importance analysis of this study showed that proximity to transportation infrastructure (road, road intersection, and rail) heavily influences IDS. This finding supports the “convenience theory” of illegal dumping, which suggests that people select disposal sites primarily based on ease of access [64]. The second highest importance of drains for IDS shows that people often use drains for waste disposal. A study by Papageorgiou et al. (2009) found that drains are often used for illegal dumping in areas with limited waste management infrastructure [52]. The higher importance of building indicates that the built environment also influences illegal dumping behaviors. The importance of STSs as a predictor presents an interesting perspective. While these facilities are designed to facilitate waste disposal, their presence correlates with IDSs. This situation might be explained by the “overflow hypothesis,” wherein inadequate capacity or irregular collection services at STSs lead to excess waste being disposed of nearby [65]. The distribution of the 100 STSs with varying designs (containers, open, in-house) and capacities suggests that the effectiveness of these facilities varies considerably. Moniruzzaman et al. (2011) also mentioned that waste is often dumped near the waste bin [66]. The importance of population density suggests that the variation in population density across KCC does not directly relate to IDS risk. These findings align with the growing recognition that ML models can play a transformative role in environmental risk detection and management [19].

The risk distribution shows that the highest risk is concentrated along road networks and urban centers. This pattern suggests that IDS is closely connected with accessibility and the built environment. This finding has important implications for waste management policies. First, it suggests that central urban areas require more intensive waste collection services and monitoring than peripheral areas. Second, it indicates that waste management planning should consider urban form, transportation networks, and infrastructure.

Many cities in developing countries lack proper databases and have limited institutional capacity for continuous monitoring. In these conditions, ML models provide a cost-effective method for IDS risk assessment. Analysis of the seven ML algorithms across multiple metrics reveals substantial performance differences. RF consistently outperformed other models across all weighting schemes. Fernández-Delgado et al. (2014) evaluated 179 classifiers and found RF consistently performing better than others [67]. Similarly, Couronné et al. (2018) demonstrated RF’s robustness across various classification tasks [68]. KNN demonstrated the weakest overall performance. Tan et al. (2016) stated that KNN generally underperforms compared to ensemble methods [69]. However, Shwartz-Ziv and Armon (2022) found that properly tuned deep learning models can outperform traditional ensemble methods [70]. Future research should focus on model optimization through hyperparameter tuning that might generate even better results than RF.

While the study provides a robust framework, there is significant scope for improvement in the methodology. The study relies on ML models trained on available datasets, which may not fully capture the nature of illegal dumping activities. Both the PU learning assumption and the reliance on secondary datasets may cause label noise and measurement error. Although PU bagging and spatial cross-validation reduce variance, uncertainty quantification at the pixel level remains an area for future improvement. Future research could integrate real-time monitoring systems and drone surveillance to improve detection accuracy. The framework was developed and validated specifically for Khulna City. Applying the framework to other cities would require context-specific predictor selection and recalibration. Additionally, the study does not explore the socio-economic factors of illegal dumping, for example, lack of waste collection services and weak law enforcement. Incorporating these factors could provide deeper insights into the main causes of IDS. Lastly, the resolution of satellite imagery used for detecting IDSs may limit the ability to identify smaller dumping areas. Utilizing higher-resolution imagery and combining it with ground data could further refine the detection process and improve the overall effectiveness of the framework.

The findings of this study also contribute to the Sustainable Development Goals (SDGs). This study supports SDG 11 (Sustainable Cities and Communities) through cleaner and safer neighborhoods, SDG 3 (Good Health and Well-Being) by lowering health risks from unmanaged waste, and SDG 6 (Clean Water and Sanitation) by reducing pollution in drains and water bodies. Overall, the proposed GeoAI-based framework provides a practical tool for identifying illegal dumping risks and supporting waste management planning.

## Conclusion

Identifying potential IDSs is a precondition for planning and infrastructure investment in MSW management. This study provides significant insights into detecting illegal dumping using a GeoAI framework in Khulna City, Bangladesh. The city lacks a proper database and monitoring system. Under similar data-scarce conditions, ML models are more effective for IDS detection than conventional approaches. The proposed framework can also be applied in other cities to detect IDSs at the community level by incorporating contextual variables. This study successfully identified high-risk areas with considerable accuracy. The findings show that proximity to roads, drains, and transportation infrastructure are the strongest predictors behind illegal dumping. On the other hand, socioeconomic factors showed relatively lower importance than expected. The spatial pattern of risk suggests that waste management strategies should be designed according to urban form and infrastructure. These findings highlight the importance of incorporating spatial structure and infrastructure in waste management planning. In data-scarce cities, the proposed framework provides a practical alternative to conventional survey-based methods. Detection accuracy could be further improved by including real-time monitoring data and high-resolution imagery in future studies. Additionally, the framework can be used for risk modeling, hotspot detection, and scenario analysis to support strategic planning and sustainable environmental management.
